# Learning Traveling Solitary Waves Using Separable Gaussian Neural Networks

**DOI:** 10.3390/e26050396

**Published:** 2024-04-30

**Authors:** Siyuan Xing, Efstathios G. Charalampidis

**Affiliations:** 1 Department of Mechanical Engineering, California Polytechnic State University, San Luis Obispo, CA 93407-0403, USA; 2 Mathematics Department, California Polytechnic State University, San Luis Obispo, CA 93407-0403, USA

**Keywords:** traveling waves, solitons, peakons, compactons, separable gaussian neural networks, physics-informed neural networks

## Abstract

In this paper, we apply a machine-learning approach to learn traveling solitary waves across various physical systems that are described by families of partial differential equations (PDEs). Our approach integrates a novel interpretable neural network (NN) architecture, called Separable Gaussian Neural Networks (SGNN) into the framework of Physics-Informed Neural Networks (PINNs). Unlike the traditional PINNs that treat spatial and temporal data as independent inputs, the present method leverages wave characteristics to transform data into the so-called co-traveling wave frame. This reformulation effectively addresses the issue of propagation failure in PINNs when applied to large computational domains. Here, the SGNN architecture demonstrates robust approximation capabilities for single-peakon, multi-peakon, and stationary solutions (known as “leftons”) within the (1+1)-dimensional, *b*-family of PDEs. In addition, we expand our investigations, and explore not only peakon solutions in the ab-family but also compacton solutions in (2+1)-dimensional, Rosenau-Hyman family of PDEs. A comparative analysis with multi-layer perceptron (MLP) reveals that SGNN achieves comparable accuracy with fewer than a tenth of the neurons, underscoring its efficiency and potential for broader application in solving complex nonlinear PDEs.

## 1. Introduction

Physics-informed Neural Networks (PINNs) [1,2] have emerged as a promising data-driven approach to solving partial differential equations (PDEs) by synthesizing data and physical laws. Moreover, they have received considerable traction because they can be efficiently adapted to solving PDEs defined on domains with arbitrary geometry. Remarkable results with PINNs have been achieved across multiple domains and physical situations, such as heat transfer [3], Navier-Stokes [4] and Euler equations [5], nonlinear dynamical lattices [6,7], and medical image processing [8], to name a few.

**Figure 1:**
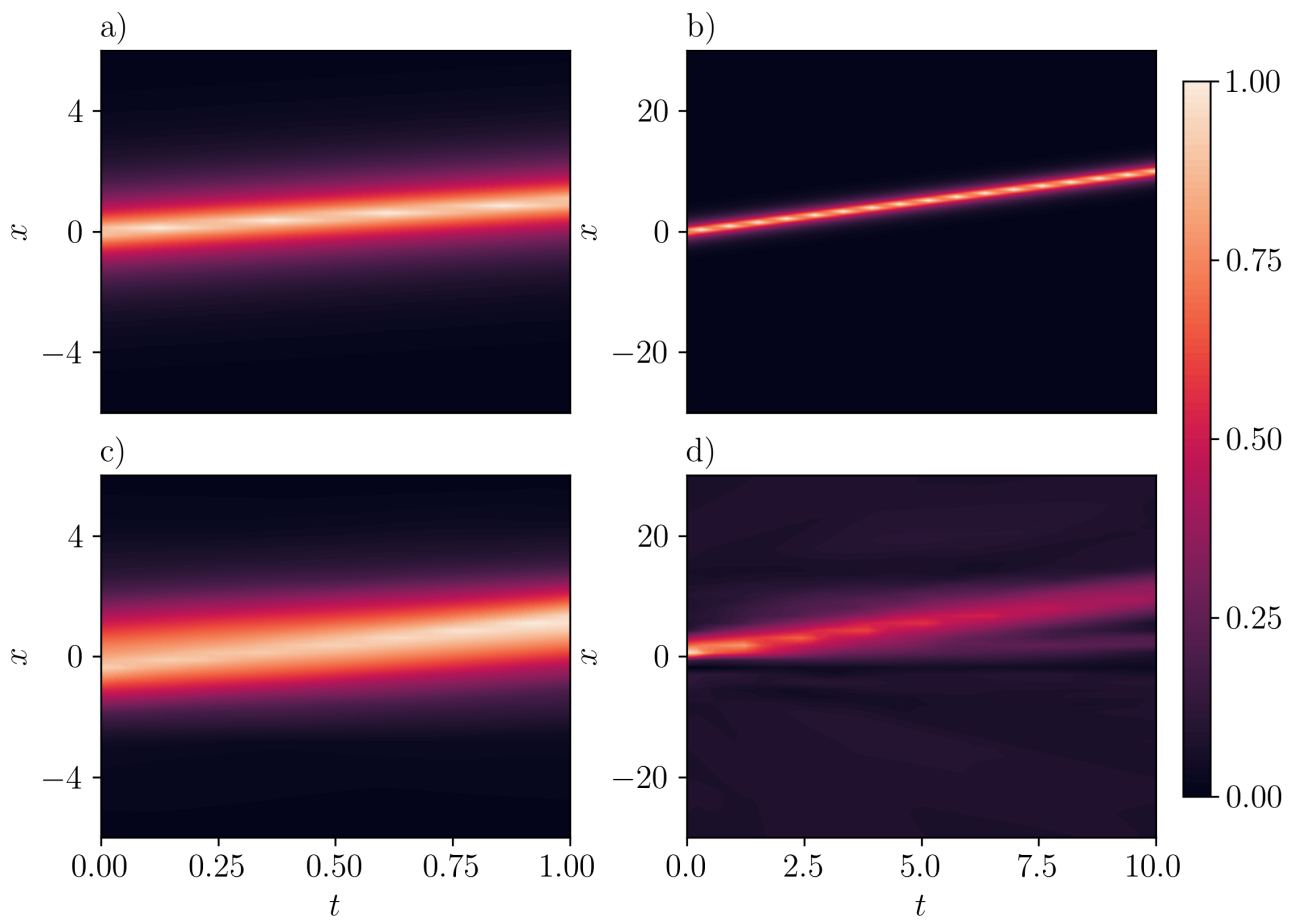
Inference of the spatio-temporal evolution of a peakon in the Camassa-Holm (CH) equation by PINNs. Panels (**a**,**b**) correspond to ground truth, whereas panels (**c**,**d**) to the NN approximation. For small domains, [−5,5]×[0,1] (panel **c**)), PINNs are able to roughly capture the correct solution. However, the propagation failure of PINNs occurs when a large spatio-temporal domain, e.g., [−30,30]×[0,10] is utilized, see, panel (**d**). In this case, PINNs converge to a trivial solution.

However, many examples of PINNs are limited to “toy” problems situated in low-dimensional spaces with small spatio-temporal, i.e., computational domains. It has been observed that PINNs often converge to incorrect or trivial solutions across a broad spectrum of problems [9,10,11] (see also [6] for a case where they fail to respect symmetries). This issue becomes more pronounced in problems with larger domains, where a phenomenon known as propagation failure [12] frequently occurs. This challenge arises because PINNs utilize an unsupervised learning scheme to solve PDEs by minimizing the residual errors of the underlying governing equations. The presence of propagation failure does not ensure convergence to a faithful solution of the physical system at hand, as numerous trivial solutions can also exhibit zero residuals. Therefore, as the learning process attempts to extend the solution from the initial and/or boundary conditions to the interior points, it often becomes “trapped” in regions of solution spaces that contain trivial solutions only. This phenomenon is particularly common when PINNs are applied to solve problems with large domains. Indicatively, Figure 1 highlights this issue in the Camassa-Holm (CH) equation [13] for a single-peakon solution (Note that the loss function of this example is modified from [14], removing the termination condition at t=T).

To address the pathologies of PINNs, multiple methods have been developed including ones that consider embedding Fourier features [15], adaptive sampling [12,16], and those respecting causality [17]. In fact, besides the physical laws embodied in PDEs themselves, the mathematical properties of their solutions can be leveraged too. For example, traveling waves (TWs) to PDEs are solutions of the form u(x±ct), where *c* is their speed (the “−” and “+” signs correspond to TWs moving, i.e., traveling to the right and left, respectively). However, a few efforts have been devoted to embedding such mathematical properties of solutions into PINNs (see, [6] for the development of symmetry-preserving PINNs) such that the output of neural networks (NNs) will automatically respect the corresponding features of the solution. This is expected to improve the efficiency in training and increase the opportunity for NNs to converge to correct solutions.

In this paper, we aim to enhance PINNs by pursuing this route. More specifically, we will focus on seeking TW solutions to nonlinear PDEs using PINNs with input transformed into a frame that co-moves with the solution, i.e., co-traveling frame. This idea has been explored in the recent work in [18] in which the characteristics of hyperbolic PDEs are encoded in the network by adding a characteristic layer. Herein, we will use this structure to learn TWs, i.e., solitary waves in multiple families of one and two-dimensional nonlinear PDEs. Those include the *b*- and ab-families of peakon equations [19,20] which contain the (completely integrable) Camassa-Holm (CH) and Degasperis-Procesi equations [13,21] (see, also [22]), and the Rosenau-Hyman compacton equations [23] (see, also [24]). In addition, a novel interpretable NN – Separable Gaussian Neural Networks (SGNNs) [25] – will be employed to extract solution forms in the sense of generalized Gaussian radial-basis functions. The description of this network will be deferred to Section 2, along with the discussion about its advantages.

The rest of the paper is organized as follows. In Section 2, we introduce the architecture of SGNN with its input transformed to a co-traveling frame. Our aim here is to integrate the mathematical description of TWs into the framework of PINNs in order to reliably identify TWs to physically relevant PDEs. In Section 3 and Section 4, we demonstrate the applicability of the method to the study of peakons in the *b*- and ab-families of peakon equations, respectively. Then, we extend this approach in Section 5 to identify 2D compacton configurations. We mention in passing that the architecture can easily predict such higher-dimensional solutions which have not been studied in the realm of PINNs, to the best of our knowledge. At last, we perform an extensive comparison of the two different architectures of PINNs with different network structures in Section 6, where the advantages and disadvantages of SGNN are discussed. We conclude our findings in Section 7, and present future research directions.

## 2. Methods

### 2.1. Architecture of SGNN for Traveling Waves

**Figure 2:**
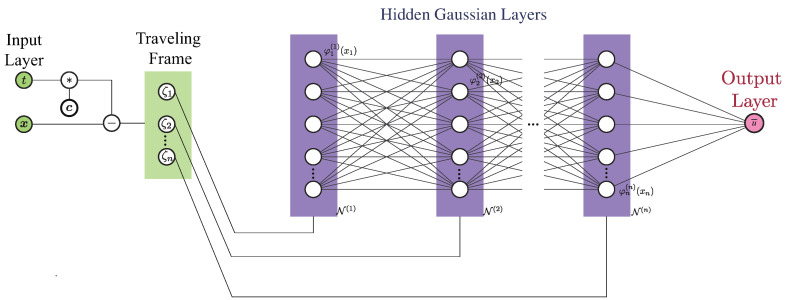
The architecture of SGNN with input transformed into the co-traveling frame whose coordinates (in vector form) are ζ=x−ct, where c represents the velocity of the wave. Different from multi-layer perceptron (MLP), the transformed input is then split and fed sequentially to hidden layers of an SGNN that consist of univariate functions. The multiplication and addition of such univariate functions in feedforward propagation can eventually lead to the summation of a set of multivariate functions used to approximate the solution of a PDE.

Inspired by [18], a *d*-dimensional (in space) TW is mapped into a frame that co-moves with it by performing the following coordinate transformation

(1) $$\zeta_{i}=x_{i}-c_{i}t,\;\;i=1,2,\cdots ,d,$$

where $c_{i}$ is its (constant) velocity in the *i*-th dimension. Under such a transformation, a TW becomes a stationary wave in the co-traveling frame. As shown in Figure 2, the coordinates $\zeta_{i}$ (i=1,2,⋯,d) become the input of the SGNN. The coordinates are then divided according to their dimensions, and sequentially fed to the feedforward layers. This results in a number of layers that is equal to the number of spatial dimensions. The neurons of each layer we consider are expressed in terms of generalized univariate Gaussian functions

(2) $$\varphi \left(\zeta_{i},\mu ,\sigma \right)=exp\left(-|\zeta_{i}{-\mu |}^{\alpha }/\sigma^{2}\right),$$

where α∈R−{0}. When α=2, φ is the regular univariate radial-basis Gaussian function. In this paper, we will adopt α=1 for peakon solutions, and α=2 for other solutions.

The first hidden layer of SGNN receives a single input: the partial coordinate $\zeta_{1}$. Subsequent hidden layers take two inputs - the output from the preceding hidden layer, and a coordinate in the traveling frame. The network culminates in a dense output layer, which aggregates the outputs from the final hidden layer. The mathematical representation of SGNN [25] is defined in the form

(3) $$\begin{array}{cc} N_{i}^{\left(1\right)} & =\varphi_{i}^{\left(1\right)}\left(\zeta_{1},\mu_{i}^{\left(1\right)},\sigma_{i}^{\left(1\right)}\right),\;1\leq i\leq N_{1}, \end{array}$$

(4) $$\begin{array}{cc} N_{i}^{\left(\ell \right)} & =\varphi_{i}^{\left(\ell \right)}\left(\zeta_{\ell },\mu_{i}^{\left(\ell \right)},\sigma_{i}^{\left(\ell \right)}\right)\sum_{j=1}^{N_{l}}W_{ij}^{\left(\ell \right)}N_{j}^{\left(\ell -1\right)},\;\;2\leq \ell \leq d,\;\;\;1\leq i\leq N_{l}, \end{array}$$

(5) $$\begin{array}{cc} \bar{u}\left(x\right) & =N\left(x\right)=\sum_{j=1}^{N_{d}}W_{j}^{\left(d\right)}N_{j}^{\left(d\right)}, \end{array}$$

where $N_{i}^{\left(l\right)}$ represents the output of the *i*-th neuron of the *l*-th layer, $N_{l}$ stands for the number of neurons of the *l*-th layer, and $\bar{u}$ is the output of SGNN. When d>2, the weights of the output layer are set to 1.

Thanks to the separable property of Gaussian radial-basis functions, the forward propagation of such univariate Gaussian functions yields the summation of multiple chains of univariate Gaussian functions, equivalent to the summation of high dimensional Gaussian radial-basis functions. In other words, the output of an SGNN equals the output of a Gaussian-Radial-Basis-Function Neural Network (GRBFNN) [26] in the form of

(6) $$\bar{u}\left(x\right)=\sum_{j=1}W_{j}G_{j},$$

where $G_{j}$ is a *d*-dimensional Gaussian radial-basis function.

The SGNN offers several advantages. Firstly, it is interpretable. The parameters of a neuron depict its local geometrical information (center and width). Without the composition of nonlinear activation functions, a human-interpretable explicit output form of Equation (6) can be obtained, in the sense of Gaussian radial-basis functions. Secondly, SGNN is easier to tune than MLP. This is because the depth of SGNN is identical to the number of dimensions; therefore, the only tunable hyperparameter is the width of each layer. Lastly, it can achieve several-order-of-magnitude more accurate results than MLP when approximating complex functions. The interested reader can consult [25] for detailed comparisons between SGNN and MLP.

### 2.2. Physics-Informed Machine Learning

The SGNN is adopted to approximate the solution u(x,t) of PDEs in the form

(7) $$u_{t}+F\left[u,u_{x},u_{xt},u_{xx},\cdots \right]=0,$$

which is subject to boundary and initial conditions (abbreviated hereafter as BCs and ICs, respectively)

(8) $$\begin{array}{cc} B[u] & =0, \end{array}$$

(9) $$\begin{array}{cc} u(x,0) & =f(x). \end{array}$$

The loss function is defined as

(10) $$L=\lambda_{r}L_{r}+\lambda_{bc}L_{bc}+\lambda_{ic}L_{ic},$$

where

(11) $$\begin{array}{cc} L_{r} & =\frac{1}{N_{r}}\sum_{i=1}^{N_{r}}{\left|R(x_{r}^{i},t_{r}^{i})\right|}^{2}, \end{array}$$

(12) $$\begin{array}{cc} L_{bc} & =\frac{1}{N_{bc}}\sum_{i=1}^{N_{bc}}{\left|B\left[u\right]\left(x_{bc}^{i},t_{bc}^{i}\right)\right|}^{2}, \end{array}$$

(13) $$\begin{array}{cc} L_{ic} & =\frac{1}{N_{ic}}\sum_{i=1}^{N_{ic}}{\left|u\left(x_{ic}^{i},0\right)-f\left(x_{ic}^{i}\right)\right|}^{2}, \end{array}$$

and $\lambda_{r}$, $\lambda_{bc}$, $\lambda_{ic}$ are scaling factors. Here, $L_{r}$, $L_{bc}$, and $L_{ic}$ represent the MSE (mean-squared error) of PDEs, BCs, and ICs, respectively. The collocation points denoted as $\left\{x_{r}^{i},t_{r}^{i}\right\}$ and $\left\{x_{bc}^{i},t_{bc}^{i}\right\}$ are randomly sampled in the domain and on the boundary, respectively. In addition, $\left\{x_{ic}^{i}\right\}$ are spatial points sampled at t=0. Throughout this paper, $\lambda_{r}$ is fixed to 1.

### 2.3. Training Scheme

In the 1D problems presented next, we employ a two-stage training process, initially using the ADAM optimizer [27] followed by the L-BFGS algorithm [28]. This approach allows us to leverage the L-BFGS algorithm’s capability to enhance convergence accuracy after the preliminary optimization with ADAM. In contrast, and for the 2D problems at hand, we solely rely on the ADAM optimizer due to the computational demands of running L-BFGS with larger datasets. The training dataset is randomly sampled using the ’Sobol’ method, which empirically can yield better results [16]. The validation dataset is created through a method of even partitioning across the domain and boundaries, ensuring comprehensive coverage and testing of the model’s predictive capabilities. Throughout the training phase, the SGNNs’ weights are initialized based on a uniform random distribution. Additionally, the initial centers of the univariate Gaussian neurons are distributed evenly across the respective dimensions, with their initial widths defined by the distance between adjacent centers.

## 3. Peakons in *b*-Family

The first model-PDE we consider in this work is the well-known *b*-family of peakon equations:

(14) $$u_{t}-u_{xxt}+\left(b+1\right)uu_{x}=bu_{x}u_{xx}+uu_{xxx},$$

that was introduced in [19]. It has been proposed as a model for the propagation of shallow water waves [19] with the parameter *b* related to the Kodama transformation group of shallow water water equations [29,30]. Moreover, Equation (14) contains two completely integrable models for b=2 and b=3, known as the Camassa-Holm equation [13,31] (see, also [22]) and Degasperis-Procesi equation [21], respectively.

The striking feature of the *b*-family of Equation (14) is that it possesses explicit single-peakon

(15) $$u\left(x,t\right)=ce^{-|x-ct|},$$

and multi-peakon solutions

(16) $$u\left(x,t\right)=\sum_{j=1}^{N}p_{j}\left(t\right)e^{-|x-q_{j}\left(t\right)|},$$

for all values of *b*, where $q_{j}$ and $p_{j}$ are the position and amplitude of *j*-th peakon with *N* representing the number of peakons, i.e., j=1,⋯,N. The peakon solution of Equation (15) (and similarly, its multi-peakon version) is not differentiable at its center, rendering its analytical and numerical study (from the PDE point of view) an extremely challenging task (see, [32] for the spectral stability analysis of peakons). It should be noted in passing that alongside the existence of peakon solutions, the *b*-family possesses explicit stationary solutions known as “leftons” [32] (and references therein) given by

(17) $$u=A(\cosh{\left(\gamma \left(x-x_{0}\right)\right))}^{-\frac{1}{\gamma }},\;\;\;\gamma =-\frac{b+1}{2},$$

where *A* and $x_{0}$ are their amplitude and center, respectively. These solutions also emerge numerically upon propagating Gaussian initial data to Equation (14) for b<−1. Even more, the propagation of Gaussian initial data to the *b*-family with −1<b<1 results in the emergence of self-similar solutions known as “ramp-cliffs”, see [32], and references therein for details.

Having introduced the model of interest, we will use the SGNN to approximate both one-peakon and multi-peakon solutions in the next section.

### 3.1. Single Peakon

#### 3.1.1. Camassa-Holm (b=2)

We first inspect a one-peakon/one-antipeakon solution in the Camassa-Holm (CH) equation. The computational domain we consider is Ω={(x,t):[−20,20]×[0,10]}. We adopt periodic BCs, and ICs of the form $u\left(x,0\right)=e^{-|x|}$ (i.e., c=1). For our analysis, we employ a one-layer SGNN with 60 neurons. As both centers and widths are trainable, the total number of trainable parameters is 180.

**Figure 3:**
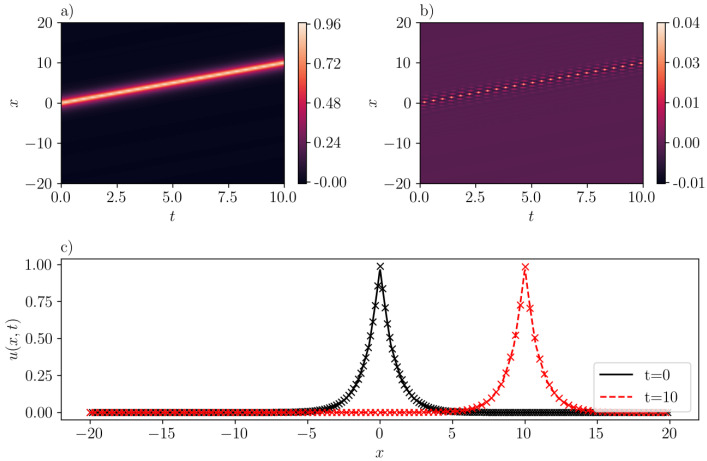
A one-peakon solution in the CH equation (b=2) with c=1. (**a**): $\bar{u}\left(x,t\right)$ inferred by SGNN; (**b**): error $e\left(x,t\right)=u\left(x,t\right)-\bar{u}\left(x,t\right)$; (**c**): $\bar{u}\left(x,t\right)$ at two time instants. In (**c**), “x” markers represent the exact solution while lines represent the prediction by SGNN. The training loss is 8.43e−3, with $\lambda_{ic}=1000$, $\lambda_{bc}=1$. Validation error: ${∥e∥}_{\infty }=3.90e-2$, ${∥e∥}_{2}=7.21e-6$.

The data collection process involves the sampling of $2^{12}=4096$ points within the specified domain. Additionally, we use the ’Sobol’ sampling scheme to gather $2^{9}=512$ boundary points, and another 512 spatial points satisfying the initial condition. It should be noted that the number of samples is larger than the number reported in the literature. This increase in sample size is attributed to the comparatively larger domain size in our analysis. We train SGNN for 5000 epochs using ADAM [27], followed by L-BFGS [28] to refine the results. The dataset is divided into 8 mini-batches. The learning rate of ADAM is 1e−2 for the first 100 epochs, and 1e−3 for the rest. We report that the mean-squared loss is 8.43e−3 when training finishes. It should also be noted that this value is scaled by a relatively large scaling factor ($\lambda_{ic}=1000$) that is selected using trial and error. On the other hand, the mean-squared validation error is much smaller, with a value of 7.21e−6. The maximum absolute validation error is 3.90e−2. As illustrated in Figure 3b, the inferred peakon solution with c=1.0 accurately approximates the exact solution with the error getting maximized at the crest of the peakon. The good agreement is also demonstrated in Figure 3c, where the “x” markers stand for the exact solution [cf. Equation (15)], and line for the predicted solution by SGNN at two different instant of times (see, the legend in the figure).

**Figure 4:**
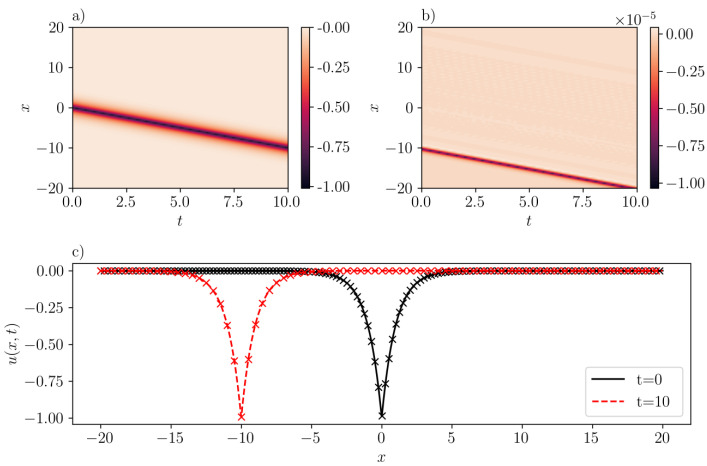
Same as Figure 3 but for an one-antipeakon solution in the CH equation (b=2) with c=−1. (**a**): $\bar{u}\left(x,t\right)$ inferred by SGNN; (**b**): error $e\left(x,t\right)=u\left(x,t\right)-\bar{u}\left(x,t\right)$; (**c**): $\bar{u}\left(x,t\right)$ at two time instants. In (**c**), “x” represents the exact solution while lines represent the prediction by SGNN. The training loss is 1.94e−11, with $\lambda_{ic}=1000,\lambda_{bc}=1$. Validation error: ${∥e∥}_{\infty }=1.02e-5$, ${∥e∥}_{2}=9.59e-13$.

A case corresponding to an anti-peakon solution with c=−1.0 is represented in Figure 4. The prediction by SGNN yields a mean-squared loss of 1.94e−11. This means that the inference of SGNN precisely matches the exact solution. The largest error occurs on the characteristic curve x+t=−10, with the magnitude level of 1e−5.

#### 3.1.2. Other Values of *b*

**Figure 5:**
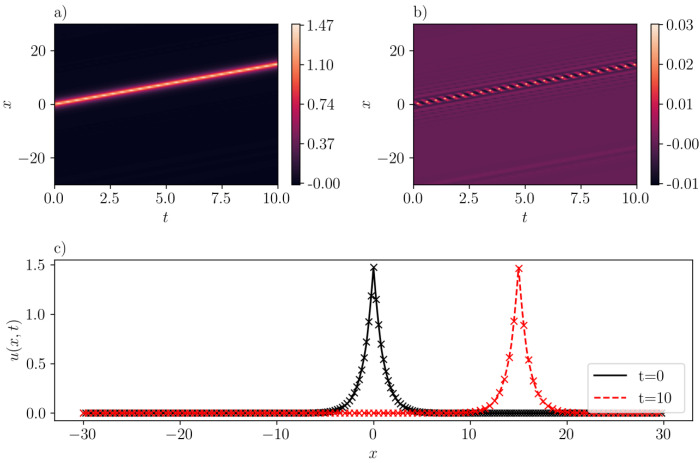
Same as Figure 4 but for a one-peakon solution of the *b*-family with b=0.8 and c=1.5. (**a**): $\bar{u}\left(x,t\right)$ inferred by SGNN; (**b**): error $e\left(x,t\right)=u\left(x,t\right)-\bar{u}\left(x,t\right)$; (**c**): $\bar{u}\left(x,t\right)$ at two time instants. In (**c**), “x” represents the analytical solution while curves represent the prediction by SGNN. The training loss is 9.18e−2, with $\lambda_{ic}=1000,\lambda_{bc}=1$. Validation error: ${∥e∥}_{\infty }=2.74e-2$, ${∥e∥}_{2}=4.09e-6$.

**Figure 6:**
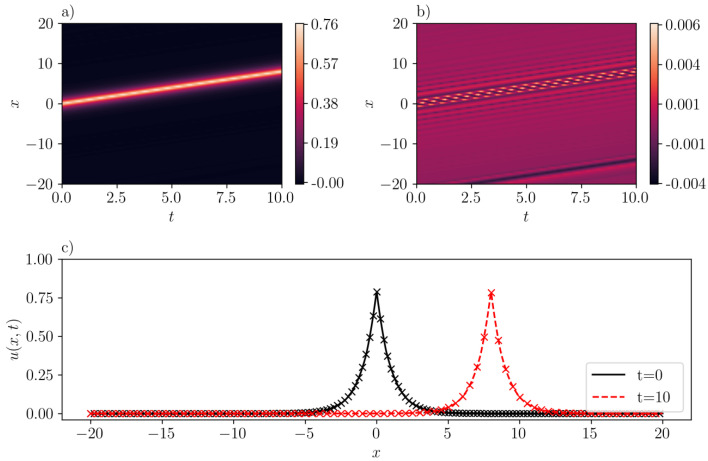
Same as Figure 5 but with b=−1.0 and c=0.8. (**a**): $\bar{u}\left(x,t\right)$ inferred by SGNN; (**b**): error $e\left(x,t\right)=u\left(x,t\right)-\bar{u}\left(x,t\right)$; (**c**): $\bar{u}\left(x,t\right)$ at two time instants. The format in panel (**c**) is the same as in panel (**c**) of Figure 5. Here, the training loss is 3.10e−2, with $\lambda_{ic}=10,000$, and $\lambda_{bc}=1$. Validation error: ${∥e∥}_{\infty }=5.92e-3$, ${∥e∥}_{2}=3.50e-5$.

We next investigate the emergence of peakons using SGNN for different values of *b*. Indeed, Figure 5a presents a peakon solution predicted by SGNN with b=0.8 and c=1.5. While the temporal domain remains as [0,10], the spatial domain is enlarged to [−30,30] in order to accommodate the rise of velocity, and thus the peakon “fits” in the computational domain over its propagation. As shown in Figure 5b, the prediction matches very well with the exact solution. The mean-squared validation error is 4.09e−6, and the maximum absolute error is 0.0274. The maximum absolute error appears at the region where the u(x,t) reaches its peak value. The training loss after 5000 epochs is reduced to 9.18e−2. The waveforms at t=0 and t=10 are depicted in Figure 5c, where lines represent SGNN’s prediction, and “x” markers represent the exact solution, respectively. The predicted peakon solution with b=−1,c=0.8 is presented in Figure 6. Likewise, a good agreement between inference by SGNN and the exact solution $u\left(x,t\right)=0.8e^{-|x-0.8t|}$ is achieved, with a training loss of 3.1e−2, a mean-squared validation loss of 3.5e−5, and a maximum absolute validation loss of 5.92e−3.

#### 3.1.3. Interacting Peakons

**Figure 7:**
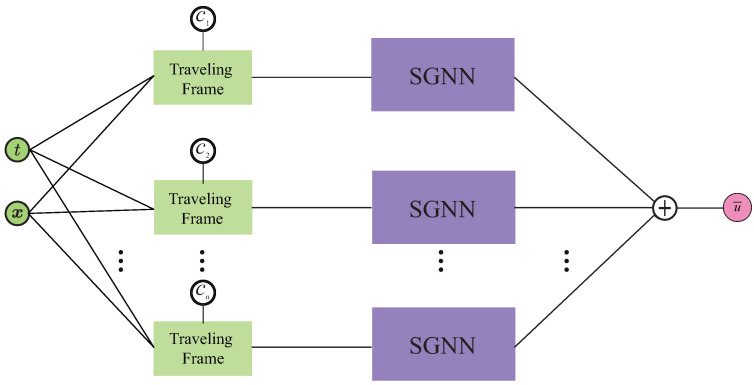
The NN architecture used for the study of multi-peakon configurations.

Having discussed the prediction of single-peakon (and anti-peakon) solutions in the *b*-family, we now turn our focus to cases involving two-peakon configurations in the CH equation (b=2), thus emulating their interactions. In particular, we focus on the following three specific scenarios: (1) peakons traveling along the same direction with identical speed, (2) peakons traveling in the same direction but at different velocities, and (3) peakons moving in opposite directions. Given that peakons can travel at varying speeds and in distinct directions in space (i.e., either left or right), we employ multiple SGNNs to approximate these peakons, allocating one SGNN per peakon. The sum of such SGNNs produces the NN-solution of Equation (14), and the NN structure in this case is shown in Figure 7. During the training stage, the loss functions associated with the PDE and BCs are identical to those in Equations (11) and (12). However, it is necessary to modify the loss function of ICs such that the output of each SGNN at t=0 accurately reflects the corresponding peakon solution at t=0.

We inspect the response from t=0 to t=10, within a spatial domain [−30,30]. Two one-layer SGNNs with 40 neurons are used. Each training dataset is generated by randomly sampling $2^{13}=8192$ collocation points within the domain, and $2^{10}=1024$ points on the boundary. The dataset is then divided into 8 mini-batches. The results are obtained with 5000 training epochs by ADAM, followed by refinement by L-BFGS (as before). The validation set is generated by uniformly sampling a 50 × 100 grid in the domain including BCs and ICs.

**Figure 8:**
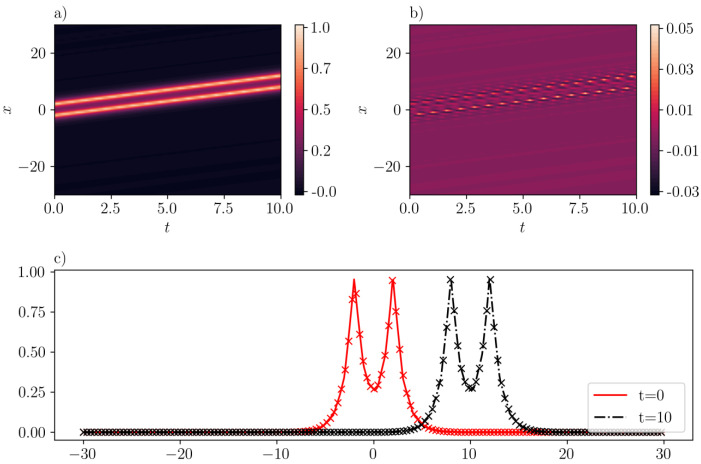
Two peakons with identical traveling speed (c=1) in the CH equation. (**a**): $\bar{u}\left(x,t\right)$ inferred by SGNN; (**b**): error $e\left(x,t\right)=u\left(x,t\right)-\bar{u}\left(x,t\right)$; (**c**): $\bar{u}\left(x,t\right)$ at two time instants. In (**c**), “x” represents the exact solution while lines represent the prediction by SGNN. The training loss is 4.27e−3, with $\lambda_{ic}=\lambda_{bc}=100$. Validation error: ${∥e∥}_{\infty }=5.22e-2,{∥e∥}_{2}=2.99e-5$.

In Figure 8, two peakons traveling towards the right with identical speed c=1 are presented. The ICs employed here are $u\left(x,0\right)=e^{-|x+2|}+e^{-|x-2|}$, which forms a bi-nominal shape. The training error is 4.27e−3, with scaling factors $\lambda_{ic}=\lambda_{bc}=100$. As shown in Figure 8a, the peakons maintain their distance during propagation. Moreover, it can be discerned from Figure 8b that SGNN is capable of making very good predictions of such configurations. Indeed, the mean-squared and maximum absolute errors are 2.99e−5 and 5.22e−2, respectively for this case. The good agreement between SGNN and exact solutions is further demonstrated in Figure 8c, where “x” makers are for exact solutions and solid lines are predictions by SGNN.

**Figure 9:**
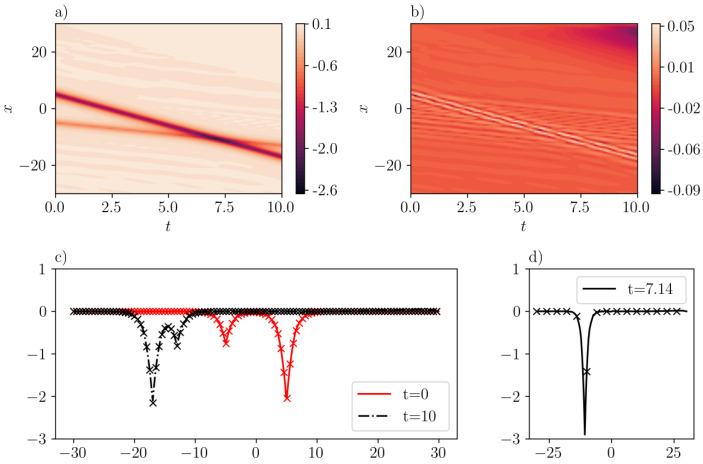
Same as Figure 8 but for the case corresponding to the interaction of two anti-peakons ($c_{1}=-0.8$, $c_{2}=-2.2$) in the CH equation. (**a**): $\bar{u}\left(x,t\right)$ inferred by SGNN; (**b**): error $e\left(x,t\right)=u\left(x,t\right)-\bar{u}\left(x,t\right)$; (**c**): $\bar{u}\left(x,t\right)$ at two time instants; (**d**): $\bar{u}\left(x,t\right)$ when two peakons collide. The format of panel (**c**) is the same as the one in Figure 8. The training loss is 1.88e−2, with $\lambda_{ic}=\lambda_{bc}=100$. Validation error: ${∥e∥}_{\infty }=9.0e-2$, ${∥e∥}_{2}=9.12e-5$.

The complementary case corresponding to the interaction of two anti-peakons traveling at different speeds is presented in Figure 9. In particular, we consider a configuration involving two anti-peakons: one centered at x=−5 with speed 0.8, and another one whose center is (symmetrically) placed at x=5 and travels with velocity of −2.2. The respective IC that describes this configuration is $u\left(x,0\right)=-0.8e^{-|x+5|}-2.2e^{-|x-5|}$. The training error is 0.0188, with scaling factors $\lambda_{ic}=\lambda_{bc}=100$. On the validation dataset, the mean-squared error is 2.99e−5. In addition, the maximum absolute error is 5.22e−2, which is reflected in Figure 9b. The interactions of these two anti-peakons are shown in Figure 9a. The second anti-peakon (in darker red), possessing a higher velocity, will eventually overtake the first one (in orange), despite initially lagging behind. A good agreement between SGNN prediction and exact solution is demonstrated in Figure 9c. The second one catches the first one at t=7.14, where their peaks add up, as shown in Figure 9d.

**Figure 10:**
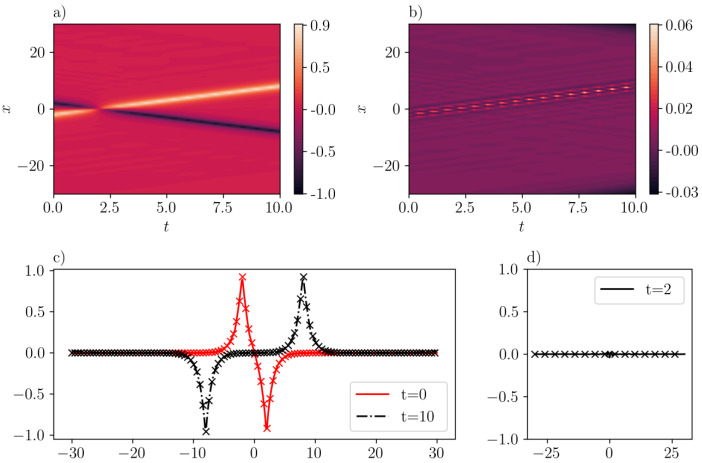
Same as Figure 9 but for the case corresponding to the “head-on” collision of a peakon and an anti-peakon (with $c_{1}=c_{2}=-1$) in the CH equation. (**a**): $\bar{u}\left(x,t\right)$ inferred by SGNN; (**b**): error $e\left(x,t\right)=u\left(x,t\right)-\bar{u}\left(x,t\right)$; (**c**): $\bar{u}\left(x,t\right)$ at two time instants; (**d**): $\bar{u}\left(x,t\right)$ when two peakons collide. The format of panel (**c**) is the same as the one in Figure 8. The training loss is 6.12e−3, with $\lambda_{ic}=\lambda_{bc}=100$. Validation error: ${∥e∥}_{\infty }=5.54e-2$, ${∥e∥}_{2}=2.11e-5$.

Finally, Figure 10 shows a more realistic scenario: the (elastic) collision between a peakon and an anti-peakon. In this case, the IC considered is given by $u\left(x,0\right)=-e^{-|x-2|}+e^{-|x+2|}$, where the training error is 6.12e−3, with scaling factors $\lambda_{ic}=\lambda_{bc}=100$. The mean-squared validation error is 2.11e−5 while the maximum absolute validation error is 5.54e−2, as illustrated in Figure 10b. As expected, the peakon (light red) and anti-peakon (in darker red) move towards each other with same velocity as shown in Figure 10a until they collide at t=2. Indeed, Figure 10d showcases the predicted solution at the time of collision where the waveforms cancel each other. Then, at later times, i.e., t>2, the anti-peakon and peakon re-emerge, and they can maintain their shape after collision, as shown in Figure 10c.

**Figure 11:**
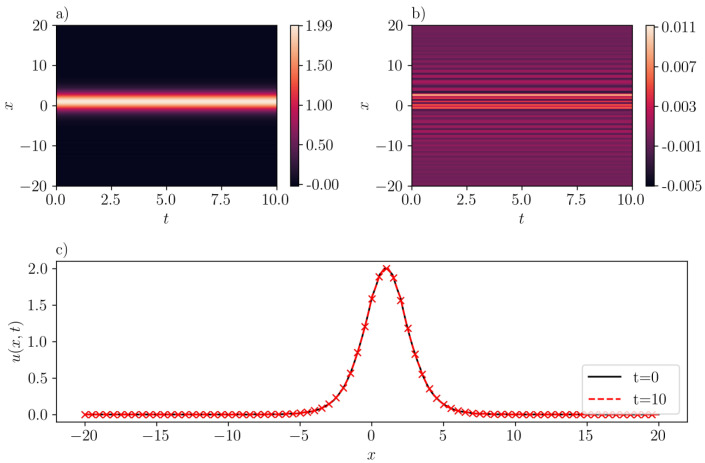
Same as Figure 10 but for a stationary solution, i.e., “lefton” of the *b*-family with b=−2.0. (**a**): $\bar{u}\left(x,t\right)$ inferred by SGNN; (**b**) $e\left(x,t\right)=u\left(x,t\right)-\bar{u}\left(x,t\right)$; (**c**) u(x,t) at two time instants. The format of panel (**c**) is the same as the one in Figure 9. The training loss is 0.054, with $\lambda_{ic}=1000$, $\lambda_{bc}=1$. Validation loss: ${∥e∥}_{\infty }=1.12e-2$, ${∥e∥}_{2}=4.62e-6$.

#### 3.1.4. Lefton Solutions

The last case that we consider using SGNNs is the lefton regime, i.e., b<−1 whose explicit solution form is given by Equation (17). Herein, we study such solutions at b=−2. For our training dataset, we randomly select $2^{12}=4096$ points within the domain alongside an additional $2^{9}=512$ points, subsequently dividing this dataset into 8 mini-batches. The chosen time domain is set at t∈[−10,10], and the spatial domain at x∈[−20,20]. As depicted in Figure 11, there is a high degree of concordance between the SGNN predictions and the exact solutions. The training loss, adjusted by scaling factors $\lambda_{ic}=1000$ and $\lambda_{bc}=1$, is recorded at 0.054. The mean-squared error for the validation loss stands at 4.62e−6, with the maximum absolute validation loss reaching 1.12e−2.

## 4. Peakons in *ab*-Family

In this section, we turn our focus on the applicability of SGNN to the so-called ab-family [20]

(18) $$u_{t}+u^{2}u_{x}-au_{x}^{3}+D^{-2}\partial_{x}\left[\frac{b}{3}u^{3}+\frac{6-6a-b}{2}uu_{x}^{2}\right]+D^{-2}\left[\frac{2a+b-2}{2}u_{x}^{3}\right]=0$$

of peakon equations where $D^{-2}$ stands for the nonlocal operator ${\left(1-\partial_{x}^{2}\right)}^{-1}$. The ab-family is a generalization of the *b*-family [cf. Equation (14)] in the sense that it corresponds to cubic (in its nonlinearity) CH-type equations unlike the quadratic CH-type equations of the *b*-family [20]. Interestingly, the ab-family admits the one-peakon solution taking the form

(19) $$u\left(x,t\right)=\pm \sqrt{c}e^{-|x-(1-a)ct|}.$$

For the applicability of SGNN, we inspect the peakon solution in the spatial domain x∈[−20,20] and time domain t∈[0,10]. A SGNN with 80 neurons is used to approximate the one-peakon solution in the ab-family. To generate the training dataset, we randomly generate $2^{13}=8192$ collection samples within the domain and $2^{10}=1024$ samples on the boundary. The training dataset is evenly split into 8 mini-batches. In the loss function, $\lambda_{ic}=1000$ and $\lambda_{bc}=100$ are applied to penalize ICs and BCs. Same as before, the ADAM method is then used to train the SGNN, followed by the refinement by L-BFGS.

**Figure 12:**
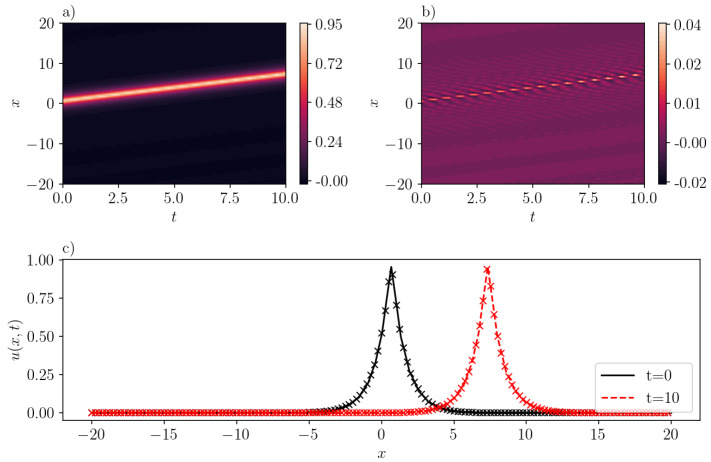
A peakon in the ab-family with b=2.0, a=1/3. The wave speed is c=1. (**a**): $\bar{u}\left(x,t\right)$ inferred by SGNN; (**b**) $e\left(x,t\right)=u\left(x,t\right)-\bar{u}\left(x,t\right)$; (**c**) u(x,t) at two time instants. In (**c**), “x” markers represent the exact solution while lines depict the prediction by SGNN. The training loss is 0.0141, with $\lambda_{ic}=1000$, $\lambda_{bc}=100$. Validation loss: ${∥e∥}_{\infty }=3.70e-2$, ${∥e∥}_{2}=8.63e-6$.

**Figure 13:**
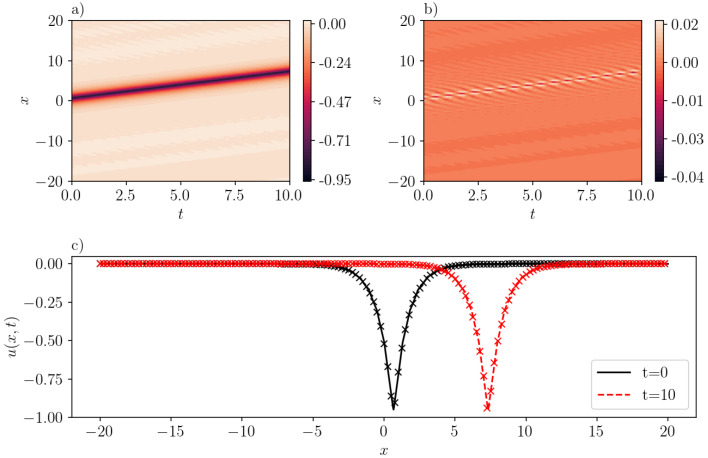
Same as in Figure 12, but for an anti-peakon in the ab-family with b=2.0, a=1/3, and wave speed c=−1. (**a**): $\bar{u}\left(x,t\right)$ inferred by SGNN; (**b**) $e\left(x,t\right)=u\left(x,t\right)-\bar{u}\left(x,t\right)$; (**c**) u(x,t) at two time instants. The format of panel (**c**) is the same as the one in Figure 12. Here, the training loss is 0.0138, with $\lambda_{ic}=1000$, $\lambda_{bc}=100$. Validation error: ${∥e∥}_{\infty }=4.05e-2$, ${∥e∥}_{2}=8.24e-6$.

Distinct from the members of the *b*-family, both peakons and anti-peakons of the ab-family propagate in the same direction. This behavior is confirmed using parameters b=2.0, a=1/3, and c=1, as illustrated in Figure 12 and Figure 13. The training losses for the peakon and anti-peakon solutions are recorded at 0.0141 and 0.0138, respectively. For the peakon solution, the mean-squared error across the validation set is measured at 8.63e−6, with the maximum absolute error reaching 3.7e−2. Similarly, the anti-peakon solution exhibits a mean-squared error of 8.24e−6 over the validation set, and its maximum absolute error is noted as 4.5e−2.

## 5. 2D Compactons

In this section, we depart from the previous one-dimensional (in space) studies, and apply SGNN in order to predict TWs in two-dimensional nonlinear wave equations. More specifically, we focus on TWs that have compact support which are referred to as compactons, and introduced in [24]. Following the notation in [24], there exists a family of PDEs denoted as $C_{N}\left(m,a+b\right)$ given by

(20) $$u_{t}+{\left(u^{m}\right)}_{x}+\frac{1}{b}{\left[u^{a}\left({▿}^{2}u^{b}\right)\right]}_{x}=0,$$

that possesses compacton TWs with m≥max(1,a−1), b>0. Here, $C_{N}\left(m,a+b\right)$ represents a *N*-dimensional compacton (with N=1,2,3) with a parameter set {*m*, *a*, *b*}. In the following, we restrict ourselves to N=2, and concentrate on the single compacton case. In other words, the network structure of Figure 2 will be used. According to [23], Equation (20) supports traveling compactons traversing in the *x* direction. In this case, we have

(21) s=x−λt,

where λ is the velocity of the compacton. The case with $C_{2}\left(m=1+b,1+b\right)$ yields an explicit solution in the form

(22) $$u=\lambda^{1/b}{\left[1-\frac{F(R)}{F(R^{*})}\right]}^{1/b},\;0<R\leq R^{*},$$

where *u* vanishes elsewhere (i.e., compact support). In Equation (22), $R=\sqrt{s^{2}+y^{2}}$, and $F\left(R\right)=J_{0}\left(\sqrt{b}R\right)$ where $J_{0}$ is the zeroth-order Bessel function, and $\sqrt{b}R^{*}$ is the root of the first-order Bessel function.

**Figure 14:**
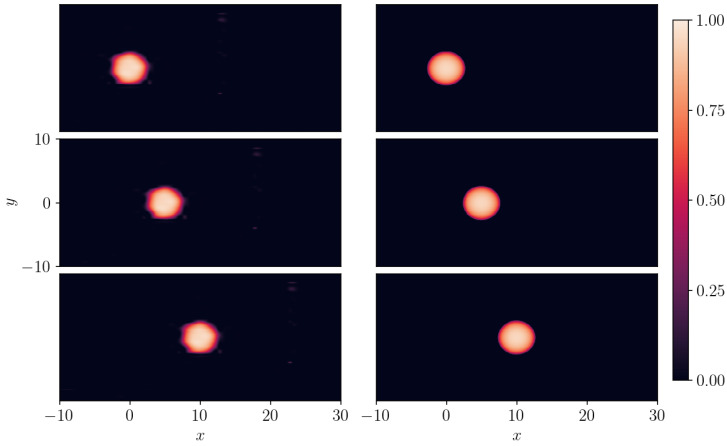
A 2D compacton $C_{2}\left(3,1+2\right)$ of Equation (22) with λ=1. Left panel: SGNN prediction; right panel: ground truth. Top panel: t=0; middle panel: t=5; bottom panel: t=10. The training loss:9.97e−3:$\lambda_{ic}=100$, $\lambda_{bc}=10$. Validation error: ${∥e∥}_{\infty }=0.371$, ${∥e∥}_{2}=1.58e-4$.

**Figure 15:**
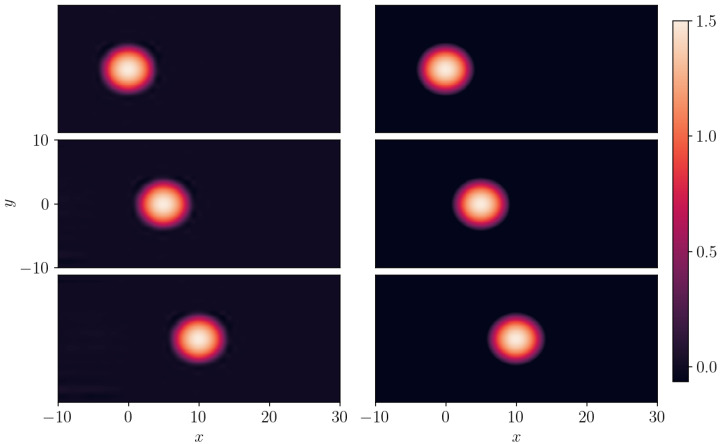
Same as Figure 14 but for the 2D compacton $C_{2}\left(2,0+3\right)$ of Equation (24) with λ=1. Left panel: SGNN prediction; right panel: ground truth. Top panel: t=0; middle panel t=5, bottom panel: t=10. The training loss:1.23e−3:$\lambda_{ic}=100$, $\lambda_{bc}=10$. Validation error: ${∥e∥}_{\infty }=0.135$, ${∥e∥}_{2}=8.28e-5$.

We use a SGNN to approximate the compacton $C_{2}\left(3,1+2\right)$. The SGNN has two layers, with 50 neurons per layer, and the approximation is performed in the spatial domain x∈[−10,30] and time domain t∈[0,10]. To generate the training dataset, we randomly sampled $2^{16}=65,536$ collocation points within the domain, along with $2^{12}=4096$ points on the boundary. The dataset is then evenly split into 8 mini-batches. The mini-batch ADAM is used to SGNN, with loss functions to minimize the residual error of the PDE, ICs, and BCs.

In Figure 14, the SGNN’s prediction (left column) is presented against the exact (right column) compacton solution $C_{2}\left(3,1+2\right)$. The training loss is stopped at 9.97e−3, with $\lambda_{ic}=100$, and $\lambda_{bc}=10$. The mean-squared validation error is 1.58e−4 while the maximum absolute error is 0.371. The compacton travels along *x*-axis with a velocity of λ=1. At t=0 (top panel), the compacton commences with a center placed at x=0. Middle and bottom panels present snapshots of compactons at t=5, and t=10, respectively. We report that the SGNN’s prediction captures the main characteristics of the exact solution although minor errors appear around the edges of compacton.

As a last case, we consider the compacton $C_{2}\left(m=2,a+b=3\right)$ whose explicit solution is given by

(23) $$u=\kappa_{N}\left[\lambda A_{N}-bR^{2}\right],0<R\leq R_{*}\equiv \sqrt{\lambda A_{N}/b}$$

with *u* vanishing elsewhere. According to Ref. [23], we pick N=2, m=0, a=0, and b=3, and thus we have

(24) $$C_{N}\left(2,0+3\right):A_{2}=\frac{3}{2}{\left(4+2\right)}^{2},\kappa_{2}^{-1}=6\left(4+2\right).$$

The SGNN’s prediction and exact solution of $C_{2}\left(2,0+3\right)$ compacton solutions are presented in the left and right columns of Figure 15. Snapshots of the solutions are shown at t=0 (top), t=5 (middle), and t=10 (bottom) therein. In this case, we report that the predictions precisely match the exact solution at these times. The scaling factors in the loss function are $\lambda_{ic}=100$ and $\lambda_{bc}=10$, and the training loss is stopped at 1.23e−3 after 300 epochs. The mean-squared validation error is 8.28e−5, while the maximum absolute error is 0.135.

In summary, we present the training losses and validation errors of all previous results (see, also the reference Figure in the left column) in Table 1.

## 6. Comparison and Discussion

To compare the performance of the traditional and new structures of PINNs, we use them to approximate a peakon solution in the CH equation with c=1. The spatial domain employed is [−20,20], while the temporal domain is [0,10]. The size of the training set is $2^{12}=4096$, with $2^{9}=512$ samples for the ICs and BCs. The selection of width and depth for models is informed by the configurations reported in existing literature. Additionally, we also compare the performance of SGNN vs. MLP. As shown in Table 2, in the traditional PINN framework, neither SGNN nor MLP can successfully converge to a TW on a large spatial and temporal domain. Despite small training losses, all NN structures get stuck at the trivial solution as it is very difficult to overcome propagation failure when dealing with enlarged domains. By introducing a training method that respects causality [17] or performs adaptive sampling [16], one may be able to address this failure.

On the other hand, as illustrated in Table 3, with the TW coordinate transformation, identical NN structures of SGNN and MLP with ReLu function all converge to the correct solution. Although the training losses of MLP with hyperbolic tangent and sigmoid functions are relatively large, they all capture the characteristics of the TW. Sigmoid and hyperbolic functions have difficulties approximating the non-differentiable peak of the peakon, with about 0.3 error in the peak value. Notably, these results can be improved by modifying the sampling method, training scheme, and loss functions. With the increase of depth and width, MLP with ReLU and sigmoid functions can further reduce loss values. The loss values with SGNN also gradually drop as width increases. SGNN excels at the compact structure that only requires less than 1/10 of training parameters. In addition, SGNN can give an explicit solution form of the PDEs in the sense of Gaussian radial-basis functions. However, increasing further the number of neurons in SGNN does not dramatically result in loss-value reduction. This could be remedied by modifying the training and sampling schemes.

Why can the modified structure of PINNs avoid propagation failure and lead to better results? We attempt to answer this question next. By mathematically transforming the NN input to the traveling coordinate x−ct, we inherently produce an output in the form of u(x−ct). This representation naturally aligns with the solution form of TWs, maintaining the integrity of the solution’s structure. From a physical perspective, this transformation converts a dynamic problem into a static one (i.e., a TW becomes stationary in a frame that co-moves with the solution), thus simplifying the problem considerably. Algorithmically, this transformation effectively reduces the input dimension by one, which can lead to a decrease in the required data size for training. Furthermore, the functional form of TWs of u(x−ct) ensures that any combination of spatial and temporal coordinates resulting in the same traveling frame coordinate will produce identical outcomes. This characteristic automatically propagates initial and/or boundary conditions along the characteristic path x−ct, significantly reducing the challenge of extending solutions from ICs and BCs to interior points.

## 7. Conclusions

In this work, we introduce a modified structure of PINNs that incorporates the mathematical description of TWs to nonlinear PDEs. In particular, we integrated a novel neural network architecture, called SGNN, into the PINNs framework. Our approach demonstrated a significant improvement in overcoming the propagation failure of PINNs, particularly in large-domain applications. Utilizing this enhanced network, we successfully generated interpretable predictions for TWs across various PDE families including the *b*- and ab-families of peakon equations. To the authors’ best knowledge, this is also the first study on applying PINNs to identify 2D TWs, such as compactons, as well as to study the collisions of 1D multiple peakons. This work opens up new directions for future studies that we plan to undertake herein. Specifically, and on the one hand, there exist solutions to nonlinear dispersive PDEs that self-similarly blow-up in finite (or infinite) time [33]. Under a stretching transformation [33], such solutions can appear as steady ones in a frame that “co-explodes” with the solution [34,35], thus enabling the applicability of the present NN architecture for the identification and prediction of self-similar collapse. On the other hand, the present NN structure could be expanded in order to model and predict the transient behavior of TWs. In addition, regularization techniques can be incorporated to refine the model to capture the essential features of the solutions more succinctly.

## Figures and Tables

**Table 1:** The training losses and validation errors of the presented results in Section 2, Section 3, Section 4 and Section 5.

Figure	Training Loss	Validation Error	
		${∥e∥}_{2}$	${∥e∥}_{\infty }$
3	8.43e−3	7.21e−6	3.90e−2
4	1.94e−11	9.59e−13	1.02e−5
5	9.18e−2	4.09e−6	2.74e−2
6	3.10e−2	3.50e−5	5.92e−3
8	4.27e−3	2.99e−5	5.22e−2
9	1.88e−2	9.12e−5	9.00e−2
10	6.12e−3	2.11e−5	5.54e−2
11	5.40e−2	4.62e−6	1.12e−2
12	1.40e−2	8.63e−6	3.70e−2
13	1.38e−2	8.24e−6	4.05e−2
14	9.97e−3	1.58e−4	3.71e−1
15	1.23e−3	8.28e−5	1.35e−1

**Table 2:** Comparison of SGNN and MLP with the traditional PINNs. Despite small loss values, no network structure can converge to the correct solution. Spatial domain: [−30,30], time domain: [0, 10]. GRB: generalized Gaussian radial-basis function.

Network	Activation	Depth	Width	Loss	Trivial Solution?
SGNN	GRBF	1	40	(2.69±5.47)e−7	Yes
MLP	ReLu	2	40	(1.13±0.60)e−3	Yes
	ReLu	4	40	(6.11±4.10)e−4	Yes
	ReLu	6	40	(1.05±0.66)e−3	Yes
	ReLu	8	20	(1.06±0.77)e−3	Yes
	sigmoid	2	40	(7.37±2.10)e−6	Yes
	sigmoid	4	40	(1.28±0.62)e−5	Yes
	sigmoid	6	40	(1.08±0.82)e−6	Yes
	sigmoid	8	20	(1.91±0.91)e−5	Yes
	tanh	2	40	(1.26±0.24)e−6	Yes
	tanh	4	40	(2.11±0.54)e−6	Yes
	tanh	6	40	(2.24±0.69)e−6	Yes
	tanh	8	20	(3.59±1.46)e−6	Yes

**Table 3:** Comparison of SGNN and MLP with PINNs incorporating with traveling frame. Spatial domain: [−30,30], time domain: [0, 10]. GRB: generalized Gaussian radial-basis function.

Network	Activation	Depth	Width	Parameters	Loss	Trivial Solution?
	GRBF	1	20	60	(1.42±0.08)e−2	No
SGNN	GRBF	1	40	120	(1.02±0.17)e−2	No
	GRBF	1	60	180	(8.65±1.22)e−3	No
MLP	ReLu	2	40	1640	(2.95±0.12)e−2	No
	ReLu	4	40	4840	(2.93±0.11)e−2	No
	ReLu	6	40	8040	(5.34±2.10)e−4	No
	ReLu	8	20	2820	(8.60±2.93)e−4	No
	sigmoid	2	40	1640	0.72±5.62e−4	No
	sigmoid	4	40	4840	0.72±6.80e−3	No
	sigmoid	6	40	8040	0.71±7.61e−3	No
	sigmoid	8	20	2820	0.72±5.33e−3	No
	tanh	2	40	1640	0.72±7.00e−3	No
	tanh	4	40	4840	0.71±4.65e−3	No
	tanh	6	40	8040	0.70±0.05	No
	tanh	8	20	2820	0.56±0.15	No

## Data Availability

The data presented in this study are available on request from the corresponding author.
